# Phosphatase PTPN22 Regulates Dendritic Cell Homeostasis and cDC2 Dependent T Cell Responses

**DOI:** 10.3389/fimmu.2020.00376

**Published:** 2020-03-04

**Authors:** Harriet A. Purvis, Fiona Clarke, Anna B. Montgomery, Chloe Colas, Jack A. Bibby, Georgina H. Cornish, Xuezhi Dai, Diana Dudziak, David J. Rawlings, Rose Zamoyska, Pierre Guermonprez, Andrew P. Cope

**Affiliations:** ^1^Faculty of Life Sciences and Medicine, Centre for Inflammation Biology and Cancer Immunology, King's College London, London, United Kingdom; ^2^Seattle Children's Research Institute, Seattle, WA, United States; ^3^Department of Pediatrics, University of Washington School of Medicine, Seattle, WA, United States; ^4^Department of Immunology, University of Washington School of Medicine, Seattle, WA, United States; ^5^Laboratory of Dendritic Cell Biology, Department of Dermatology, Friedrich-Alexander University of Erlangen, Erlangen, Germany; ^6^Centre for Immunity, Infection and Evolution, Institute of Immunology and Infection Research, University of Edinburgh, Edinburgh, United Kingdom; ^7^Centre for Inflammation Research, CNRS ERL8252, INSERM1149, Université de Paris, Paris, France

**Keywords:** dendritic cell, PTPN22, homeostasis, T follicular helper cell, proliferation, autoimmunity, polymorphism, cDC2

## Abstract

Dendritic cells (DCs) are specialized antigen presenting cells that instruct T cell responses through sensing environmental and inflammatory danger signals. Maintaining the homeostasis of the multiple functionally distinct conventional dendritic cells (cDC) subsets that exist *in vivo* is crucial for regulating immune responses, with changes in numbers sufficient to break immune tolerance. Using *Ptpn22*^−/−^ mice we demonstrate that the phosphatase PTPN22 is a highly selective, negative regulator of cDC2 homeostasis, preventing excessive population expansion from as early as 3 weeks of age. Mechanistically, PTPN22 mediates cDC2 homeostasis in a cell intrinsic manner by restricting cDC2 proliferation. A single nucleotide polymorphism, PTPN22^R620W^, is one of the strongest genetic risk factors for multiple autoantibody associated human autoimmune diseases. We demonstrate that cDC2 are also expanded in mice carrying the orthologous PTPN22^619W^ mutation. As a consequence, cDC2 dependent CD4^+^ T cell proliferation and T follicular helper cell responses are increased. Collectively, our data demonstrate that PTPN22 controls cDC2 homeostasis, which in turn ensures appropriate cDC2-dependent T cell responses under antigenic challenge. Our findings provide a link between perturbations in DC development and susceptibility to a broad spectrum of PTPN22^R620W^ associated human autoimmune diseases.

## Introduction

Dendritic cells (DCs) are specialized antigen presenting cells that sense danger signals and instruct T cell responses (1). Distinct subsets of DCs exist *in vivo*, broadly divided into conventional (cDC) and plasmacytoid (pDC) subsets. In mice, cDCs (CD11c^+^MHCII^+^) are sub-divided into functionally distinct phenotypes defined as cDC1 (CD8^+^IRF8^+^XCR1^+^Clec9a^+^CD24^+^) and cDC2 (IRF4^+^CD11b^+^SIRPα^+^) whilst in humans the equivalent DC subsets are defined by expression of CD8^+^IRF8^+^XCR1^+^Clec9a^+^CD141^+^ (cDC1) and IRF4^+^CD1c^+^ (cDC2) (1–4). Functionally, cDC1 cross-present exogenous antigens to activate CD8^+^ T cells and can promote IL-12 dependent Th1 responses (1, 5–7). In comparison, cDC2s are potent activators of CD4^+^ T cells (8). Under polarizing inflammatory conditions, cDC2 induce Th2 responses in the lung (9, 10), drive Th17 responses through IL-23 secretion (11–17), and initiate SIRPα dependent induction of T follicular helper cells (T_FH_) and germinal center (GC) formation (18).

Maintenance of cDC homeostasis is crucial for regulating immune responses, with deregulation resulting in infection and autoimmunity (19–22). This control of cDC homeostasis is mediated by a number of factors that drive cDC differentiation, proliferation, and survival or apoptosis (4). Differentiation of cDCs is initiated within the bone marrow where common DC precursors (CDP) transition to an intermediate preDC developmental stage (including pre-cDC1 and pre-cDC2s) (23–28), with terminal differentiation into cDC1 and cDC2 subsets occurring in the periphery (29); cDC1 and cDC2s are then dependent on Flt3L for their development and proliferation (29). Furthermore, local signals transduced through NOTCH2 (12, 15) or LTβR (30, 31) contribute to cDC homeostasis within specific tissue niches. Indeed, LTβR signaling is particularly important for inducing cDC2 proliferation within secondary lymphoid organs (SLOs) (30, 31).

*PTPN22* encodes a tyrosine phosphatase that negatively regulates immune receptor activation. It functions by dephosphorylating Src and Syk family kinases operating proximal to immune-receptors such as TCR, BCR, and LFA-1 (32–36). PTPN22 also operates in a phosphatase independent manner, directly binding to TRAF3 in myeloid cells and promoting type 1 interferon dependent TRAF3 ubiquitination (37). Regarding its contribution to disease, a C1858T single nucleotide polymorphism within *PTPN22* (encoding R620W) is one of the strongest genetic risk factors outside the HLA for the development of multiple autoimmune diseases, including rheumatoid arthritis, type I diabetes, and lupus (38). Investigations into the functional effects of this variant have demonstrated that *PTPN22*^R620W^ confers a missense mutation in the P1 domain of the PTPN22 PEST region, resulting in reduced binding to the negative regulatory tyrosine kinase Csk, and TRAF3 (37, 39). However, the consequence of PTPN22^R620W^ on immune function remains unclear, appearing to depend on the cellular context and signaling pathway under investigation. Indeed, both gain- and loss-of-phosphatase function effects of *PTPN22*^R620W^ have been described (40).

Using *Ptpn22* mutant mice, we describe PTPN22 as key mediator in the restriction of cDC2 populations. Perturbation of cDC2 homeostasis is phenocopied in mice carrying the human autoimmune associated variant, translating to accentuated cDC2-driven T cell responses upon antigenic challenge. Based on these data, we propose that disruption of cDC homeostasis by *PTPN22*^*R*620*W*^ genetic polymorphism contributes to the breeching of immune tolerance during the earliest phase of autoimmunity.

## Methods

### Mice

*Ptpn22*^−/−^ mice and *Ptpn22*^R619W^ mutant mice were backcrossed >10 generations to the C57BL/6 strain, their generation is described in Dai et al. (33) and Brownlie et al. (41). Mice were age and sex-matched within each individual experiment and were used at either 2–4 months or as otherwise indicated. *Ptpn22*^fl/+^ mice were bred with PC3-Cre mice and backcrossed to C57BL/6 mice for four generations and were used between 8 and 12 weeks of age. OT-II TCR transgenic CD45.1, CD45.1, and CD45.1/2 transgenic mice were used between 8 and 16 weeks of age. Where indicated, tissue was obtained, shipped on ice and processed within 24 h from mice bred and maintained at Edinburgh University under U.K. Home Office approved guidelines. Mice were age and sex-matched within each individual experiment and were used at either 2 or 6 months of age. Unless otherwise stated mice were maintained under specific pathogen free (SPF) conditions at King's College London Facility according to UK Home Office approved protocols.

### Tissue Processing

Spleens and LNs were injected with RPMI containing Liberase-TL (0.1 mg/ml; Roche) and DNase 1 (0.1 mg/ml; Sigma), and incubated at 37°C 5% CO_2_ for 30 min. EDTA (10 mM) was added for the final 5 min of the 30-min incubation. Spleen single cell suspensions were RBC lysed (Biolegend). Blood obtained by cardiac puncture was incubated at room temperature 1 h and serum separated following centrifugation. Bone marrow was flushed from the femurs and tibias of WT and *Ptpn22*^−/−^ mice, RBC lysed and pelleted. Cell suspensions were prepared from the small intestine after removal of Peyer's patches and fat. Intestines were opened longitudinally, washed of fecal contents, cut into 5 cm pieces and incubated in HBSS medium (Life Technologies) with 2 mM EDTA for 20 min at 37°C with rotation. Tissue pieces were washed in HBSS medium, minced and incubated in HBSS medium + 2% FBS with collagenase VIII (100 U/ml, Sigma, C2139) and DNAse1 (20 μg/ml) at 37°C for 40 min with rotation. Cell suspensions was passed through a 40 μm filter and pelleted at 350 g 15 min 4C. Cells were then stained for flow cytometry.

Cells from all tissues were resuspended in PBS and live cells counted by trypan blue discrimination.

### Bone Marrow Chimeras

CD45.1 or CD45.1/2 mice were hematopoietically-lethally irradiated by exposure to 9Gy for 6 min. Six hours later, bone marrow cells (2.5–5 × 10^6^ cells in 100 μl) were i.v transferred into irradiated recipients. Chimeric mice were analyzed 8 weeks after bone marrow transfer (unless otherwise indicated). As a control for complete replacement of recipient bone marrow, CD45.1^+^ recipients received 100% CD45.2^+^ C57BL/6 bone morrow.

### T Cell Adoptive Transfers

Total CD4^+^ T cells from the lymph nodes (LN) and spleens of 8–16-week old WT OT-II mice were isolated using CD4^+^ MACS negative selection kit according to manufacturer's instructions (Miltenyi Biotech). Purity of CD4^+^ T cells was determined by flow cytometry (routinely 90–95%). CD4^+^ T cells isolated from CD45.1 WT OT-II mice were labeled with cell trace violet (CTV; 2 μM; Invitrogen) at 2 × 10^7^ cells/ml in PBS for 20 min at 37°C, and excess CTV quenched in complete medium for 20 m at 37°C. Recipient mice received 0.5–1 × 10^6^ CTV^+^CD4^+^OT-II T cells resuspended in 100 μL PBS i.v. The following day T cell recipient mice were immunized i.v with 33D1-ovalbumin (200 μg/mouse) (42) in the presence or absence of sheep RBC (SRBC; Antibodies-online.com; 20 × 10^7^ cells/mouse). After 3 days spleens were assessed by flow cytometry for CTV dilution and CXCR5^+^ PD1^+^ T_FH_ within live, singlet, CD45.1^+^, CD4^+^, Vα2Vβ5^+^ cell gate.

### DC and CD4^+^ T Cell Co-culture

CD4^+^ T cells isolated from OT-II mice were labeled with cell trace violet (CTV) (2 μM; Invitrogen) at 2 × 10^7^ cells/ml in PBS for 20 min at 37°C, and excess CTV quenched in complete medium for 20 min at 37°C. T cells were co-cultured with isolated DC at a 2:1 T cell to cDC ratio 2 x 10^6^:1 x 10^6^ cells/ml in round bottom 96-well plates in the presence or absence of 33D1-ovalbumin (10 μg/ml) or anti-DEC205-ovalbumin (10 μg/ml) and cells were co-cultured for 6 days at 37°C 5% CO_2_. At day 6 cells were stained with fixable viability dye eFluor-506 (eBioscience), anti-CD3ε and anti-CD4. CTV dilution gated on live, singlet, CD3^+^CD4^+^ T cells was assessed by flow cytometry.

### BrdU Labeling

Three days prior to analysis mice were i.p injected daily with BrDU (10 mg/kg) and maintained ad libitum on drinking water containing BrdU (0.5 mg/ml).

### Flt3 Ligand Bone Marrow DC

Bone marrow was RBC lysed and cells seeded at 1 × 10^6^ cells/ml in 6-well tissue culture plates in RPMI-1640 supplemented with glutamax, 10% heat-inactivated FBS, β-mercaptoethanol (5 0 μM), penicillin/streptomycin (100 μg/ml), and HEPES 1 mM (Sigma) and Flt3L (200 ng/ml; Biolegend). Flt3L-BMDCs were cultured for 8 days at 37°C and 5% CO_2._ At day 8 non-adherent and adherent Flt3L-BMDC were harvested with trypsin-EDTA (Sigma) incubated for 2 min at room temperature. Cells were washed and live cells counted by trypan blue discrimination and seeded at 1 × 10^6^ cells/ml on 96-well flat bottom plates for 48 h in the presence or absence of anti-LTβR (2 μg/ml; 3C8; AdipoGen).

### Flow Cytometry

Fluorochrome or biotin-conjugated antibodies were used to stain single cell suspensions for flow cytometry. Fc receptors were blocked with anti-mouse CD16/CD32 (93; Biolegend) and dead cell exclusion was performed using Fixable Viability Dye (eBioscience). FACS buffer was made of PBS with 1% bovine serum albumin (Sigma) and 2 mM EDTA (Sigma). For BrDU staining following surface staining, splenocytes were fixed and permeabilised using APC-BrDU Flow Kit (BD Pharmingen). Cells were acquired using BD Fortessa or FACSCanto II flow cytometers. Performed in the Biomedical Research Center Flow Core Facility (Guy's and St Thomas' NHS Foundation Trust and King's College London). Flow cytometry gates were determined by fluorescence minus one controls. Flow cytometry analysis was performed using FlowJo software (TreeStar; 10.5.3).

### Flow Cytometry Monoclonal Antibodies Specific to Mouse

CD3ε (145-2C11), CD4 (GK1.5), CD8a (53-6.7), CD11b (M1/70), CD11c (N418), CD24 (M1/69), CD45R/B220 (RA3-6B2), CD45.1 (A20), CD45.2 (104), CD86 (GL-1), CD103 (2E7), CD172a (SIRP alpha; P84), CD279 (PD1; 29F.1A12), CXCR5 (L138D7), I-A^b^ (AF6-120.1), TCR Vα2 (B20.1), TCRVβ5 (MR9-4), CD16/32 (93), Ki67 (16A8), Ly-6G/Ly-6C (RB6-8C5), Ly-6G (1A8), (Ly6C (AL-21), Ter-119 (TER-119), NK-1.1 (PK136), CD19 (6D5), DCIR2 (33D1), cKit (2B8), Flt3 (A2-F10), LTβR (5G11) that were bought from Biolegend, eBioscience, or BD.

### Immunofluoresence Staining

Spleens were harvested into RPMI and 10% FBS and dried prior to being frozen at −80°C in OCT. Ten micrometers sections were generated and mounted onto slides using a Leica cryostat. Sections were fixed with 4% PFA for 15 min and washed with PBS. Sections were blocked for 30 min in blocking buffer (PBS + 2% FBS, rat serum (1 in 200) and anti-CD16/CD32 (1 in 200) for 30 min at room temperature. Sections were stained for 1 h at room temperature with B220-FITC and 33D1-Alexa-647 in PBS 2% FBS. Slides were washed 3 times with PBS prior to mounting in fluorescence mounting media (DAKO). Images were collected using an Olympus IX83 inverted microscope and image processing was performed using Fiji.

### Real-Time PCR

Total RNA was extracted from FACS isolated cDC2 using TRIzol reagent. Equal amounts of mRNA (determined by nanodrop; ThermoScientific) were reverse transcribed to produce cDNA using first strand cDNA synthesis using random hexamers. Gene expression was measured by SYBR Green quantitative real-time PCR using primers: *Bcl2* forward, TGAGTACCTGAACCGGCATCT, *Bcl2* reverse, GCATCCCAGCCTCCGTTAT; *Bim* forward, GGCCCCTACCTCCCTACA, *Bim* reverse, GGGGTTTGTGTTGATTTGTCA; *Trim2* forward, TTTCCATAATCACTCTGTCAAGGT, *Trim2* reverse, CCATTGGAGCCAAACTTCA; *Gapdh* forward, ACCACAGTCCATGCCATCAC *Gapdh* reverse, TCCACCACCCTGTTGCTGTA. Reactions were run using ABI Prism 7700 Sequence Detection System (Applied Biosystems). Ct values were determined with SDS software (Applied Biosystems) and gene expression levels were determined according to the dCt method (relative abundance = 2^(−dct)^ and normalized to *GAPDH* housekeeper).

### Serum Flt3L

Blood obtained by cardiac puncture was incubated at room temperature 1 h and serum separated following centrifugation. Serum Flt3 Ligand was determined by Mouse/Rat Quantikine ELISA (R&D Systems) according to manufacturer's protocol and detected using Victor 1420 multilabel counter (Perkin Elmer).

### Statistical Analysis

GraphPad Prism software was used for statistical analysis by unpaired or paired *T*-test. *P* < 0.05 were considered significant; NS = not significant, ^*^p < 0.05, ^**^ *p* < 0.01, ^***^p < 0.001, ^****^p < 0.0001.

## Results

### PTPN22 Is a Negative Regulator of cDC2 Homeostasis

**Figure 1:**
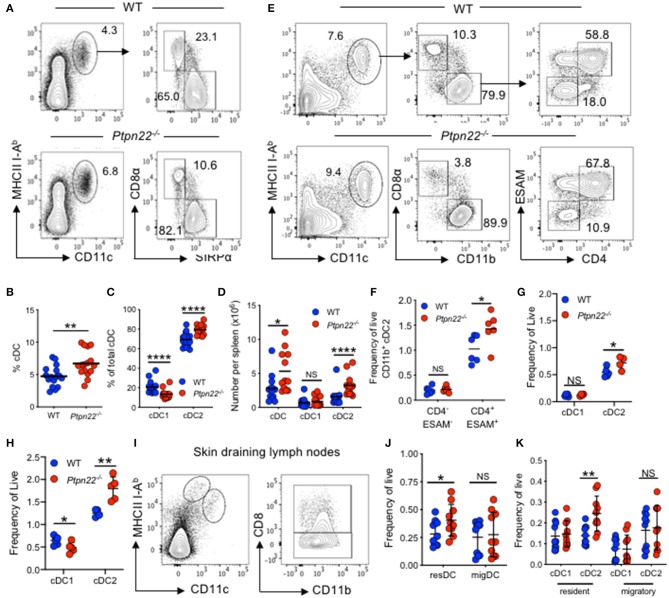
PTPN22 negatively regulates ESAM^HI^ cDC2 homeostasis. **(A–D)** Spleens of 2–4-months age matched wild type (WT) and *Ptpn22*^−/−^ mice were evaluated for cDC subsets by flow cytometry, gated on: live, singlet, lin^−^ (CD3, CD19, B220, Ly6C/G, NK1.1, Ter119), CD11c^+^MHCcII I-Ab^+^ and then CD8^+^ vs. SIRPα^+^ **(A)** Representative flow cytometry plot analysis of cDC subsets. **(B)** The proportion of CD11c^+^I-A^b+^ cDC, **(C)** the proportion of CD8^+^ cDC1 and SIRPα^+^ cDC2s, **(D)** the number of cDC, cDC1 and cDC2 per spleen. *N* = 12–15 mice per genotype from >3 independent experiments. **(E,F)** Spleens of 2–4 months age matched wild type (WT) and *Ptpn22*^−/−^ mice were evaluated for cDC subsets by flow cytometry, gated on: live, singlet, lin^−^ (CD3, CD19, B220, Ly6C/G, NK1.1, Ter119), CD11c^+^MHCcII I-Ab^+^, CD8^−^CD11b^+^, ESAM vs. CD4. Representative flow cytometry plot analysis of cDC subsets **(E)** and the frequency of CD11b ^+^DC2 ESAM^+/−^ CD4^+/−^ subsets per spleen **(F)**. *N* = 6 mice/genotype from two independent experiments. **(G)** Splenic cDC1 and cDC2 within pre-wean (3 weeks) and **(H)** post wean (4 weeks) WT and *Ptpn22*^−/−^ mice. *N* = 4 mice/genotype. **(I–K)** Lymph node resident and migratory cDC subsets within 2–4-months age matched WT and *Ptpn22*^−/−^ mice. Determined by flow cytometry gating on: singlet, live, lin^−^ CD11c^+^ MHCcII I-A^bInt^ (resident DC) or CD11c^+^ MHCcII I-A^bHigh^ (migratory DC), and then CD8α (cDC1) vs. CD11b^+^ (cDC2). **(I)** Representative flow cytometry plots. **(J)** Frequency of resident and migratory cDC and **(K)** frequency of resident and migratory cDC1 and cDC2; *N* = 10 mice/genotype from 3 independent experiments. Each point represents an individual mouse; bars represent mean, NS, not significant; ^*^p < 0.05, ^**^p < 0.01, ^****^p < 0.0001, determined by unpaired *T*-test.

*Ptpn22*^−/−^ mice have a well-characterized age dependent increase in effector/memory T lymphocytes and spontaneous GC production. However, the effect of PTPN22 on myeloid lineages is not understood. To address this, we examined if myeloid cell lineages were altered in mice lacking PTPN22. We detected a similar frequency of monocytes, macrophages, and neutrophils (Supplementary Figures 1A–C) within the spleens of WT and *Ptpn22*^−/−^ mice. In contrast, analysis of the splenic cDC compartment revealed that both the proportion and number of cDCs was increased in *Ptpn22*^−/−^ mice compared to WT (Figures 1A,B). Further phenotyping of cDC1 and cDC2 subsets revealed that the proportion of cDC2 was increased in *Ptpn22*^−/−^ mice, whereas the proportion of cDC1 was decreased (Figures 1A,C). By comparing the number of splenic cDC1s and cDC2s within WT and *Ptpn22*^−/−^ mice we found that changes in cDC subset proportions were due to selective expansion of the cDC2 subset and not loss of cDC1 cells (Figure 1D). Indeed, expansion of splenic cDC2s occurred within the *bona fide* DCIR2(33D1)^+^ESAM^+^CD4^+^CCR2^−^ cDC2 subset, whereas numbers of the “monocyte-like” DCIR2(33D1)^−^ESAM^−^CD4^−^CCR2^+/−^ DCs were similar (Figures 1E,F and Supplementary Figures 1D–F). Analyzing the kinetics of cDC2 expansion demonstrated that perturbation of cDC2 homeostasis could be detected as early as 3 weeks (Figures 1G,H), increasing further as the mice age (Supplementary Figure 1G). We confirmed these findings in WT and *Ptpn22*^−/−^ mice bred and maintained in an independent animal facility suggesting that cDC2 expansion was unlikely to be due to facility-associated environmental factors (Supplementary Figures 1H,I).

To examine if cDC homeostasis was altered in other lymphoid tissues, we examined cDC populations within skin draining lymph nodes (sdLN), and found that *Ptpn22*^−/−^ mice displayed expanded resident, but not migratory cDC2. Once again similar numbers of cDC1 were observed within both migratory and resident cDC populations (Figures 1I–K). A similar phenotype was documented within the mesenteric lymph nodes, wherein resident CD11b^+^ CD103^−^ cDC2 were expanded, while CD103^+^ resident cDCs and all migratory (IA^bhigh^) cDC subsets assessed were unaltered (Supplementary Figures 1J-L). Furthermore, in line with Ptpn22 not regulating migratory cDC2, cDC2 positioning within the spleen bridging channels appeared similar between WT and *Ptpn22*^−/−^ mice (Supplementary Figure 1M). Together, we conclude that PTPN22 regulates resident DC2 homeostasis within secondary lymphoid organs (SLOs).

### PTPN22 Regulates cDC2 Homeostasis via a DC Intrinsic Mechanism

**Figure 2:**
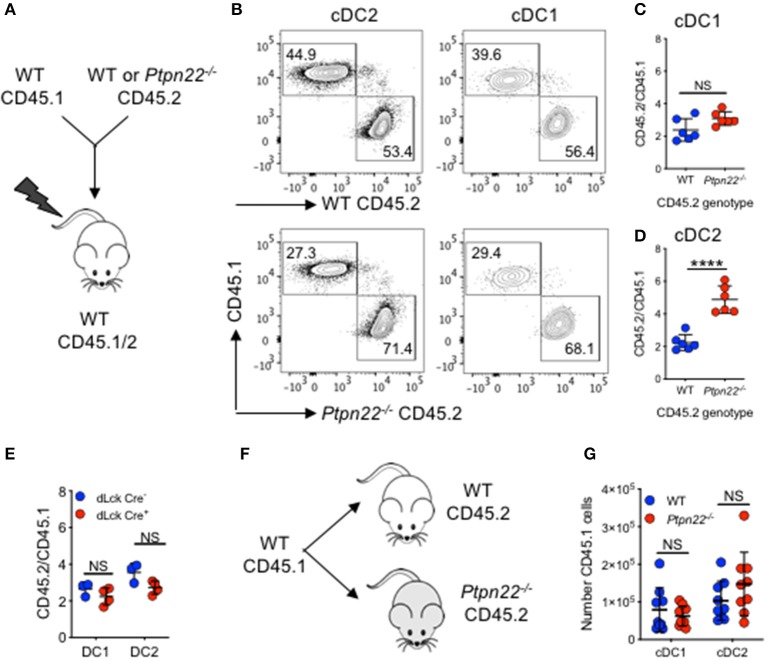
PTPN22 regulates cDC2 homeostasis in a DC intrinsic manner. **(A–D)** Lethally irradiated CD45.1/2 recipient mice received a 1:1 ratio of WT CD45.1: WT or *Ptpn22*^−/−^ CD45.2 bone marrow (i.v). After 8 weeks spleens of recipient mice were evaluated for cDC subsets and the ratio of CD45.1:CD45.2 within each subset was determined by flow cytometry gating on: live, singlet, lin^−^ CD11c^+^, MHCcII I-A^b+^ and then CD8^+^ vs. SIRPα^+^ CD45.1^+^ vs. CD45.2^+^. **(A)** Experiment schematic, **(B)** representative flow cytometry staining gated on either cDC2 (left) or cDC1 (right) subsets. **(C,D)** The ratio of CD45.1:CD45.2 within cDC1 and cDC2 subsets calculated relative to the input ratio. *N* = 5–6 mice/genotype, one experiment of two. **(E)** Lethally irradiated wild type (WT) CD45.1/2 mice received a 1:1 ratio of WT CD45.1: dLckCre^−^ or dLckCre^+^ (*Ptpn22*^−/−^*)* CD45.2 bone marrow (i.v). After 8 weeks spleens of recipient CD45.1/2 mice were evaluated for cDC subsets and the ratio of CD45.1:CD45.2 within each subset was determined by flow cytometry relative to the input ratio, *N* = 3–4 mice/genotype. **(F)** WT CD45.1 bone marrow was transferred i.v into WT or *Ptpn22*^−/−^ CD45.2 recipient mice and after 6 days the spleens of recipient mice were evaluated for the number of CD45.1 cDC1 and cDC2 by flow cytometry. **(F)** Schematic of experiment **(G)** *N* = 9 mice/genotype, two independent experiments. Each point represents an individual mouse; bars represent mean and standard deviation, NS, not significant; ^****^p < 0.0001 determined by unpaired *T*-test.

Given its broad expression in multiple hematopoietic lineages, PTPN22 has the potential to control cDC2 homeostasis through DC intrinsic or DC extrinsic mechanisms. To investigate this, we generated *Ptpn22*^−/−^ CD45.2: WT CD45.1 mixed bone marrow chimeras and found that CD45.2 *Ptpn22*^−/−^ bone marrow out-competed WT CD45.1 in the generation of cDC2 (Figures 2A–D), whereas no change was observed in the ratio of Lin^+^ cells (Supplementary Figure 2A). Consistent with our previous observations, generation of cDC1 was unaffected by genotype, further supporting a role for PTPN22 as a selective, DC intrinsic regulator of cDC2 homeostasis. PTPN22 regulates T cell homeostasis (43), raising the possibility of an indirect cDC2 phenotype driven by enhanced T cell activation in PTPN22:WT chimeras. Therefore, we examined if lineage specific deletion of *Ptpn22* within the T cell compartment would have an impact on cDC2 populations. We detected no differences in cDC2 expansion in either mice with T cell restricted *Ptpn22*^−/−^ (Supplementary Figure 2B) or between chimeras harboring PTPN22 sufficient or deficient T cells (Figure 2E), indicating that deficiency of *Ptpn22* exclusively in T cells was not sufficient to perturb cDC homeostasis.

Finally, we determined if the *Ptpn22*^−/−^ environment (via an indirect effect on stroma), contributed to cDC2 expansion. WT CD45.1 bone marrow was transferred into non-irradiated WT and *Ptpn22*^−/−^ CD45.2 mice. After 6 days we observed no difference in CD45.1 cDC2 numbers developing within either WT or *Ptpn22*^−/−^ mice (Figures 2F,G). This result is consistent with previous reports establishing that PTPN22 expression is restricted to hematopoietic cell lineages (44, 45). Together, these data suggest that PTPN22 regulates cDC2 homeostasis via a cDC intrinsic mechanism.

### PTPN22 Regulates cDC2 Homeostasis After Pre-cDC Development

**Figure 3:**
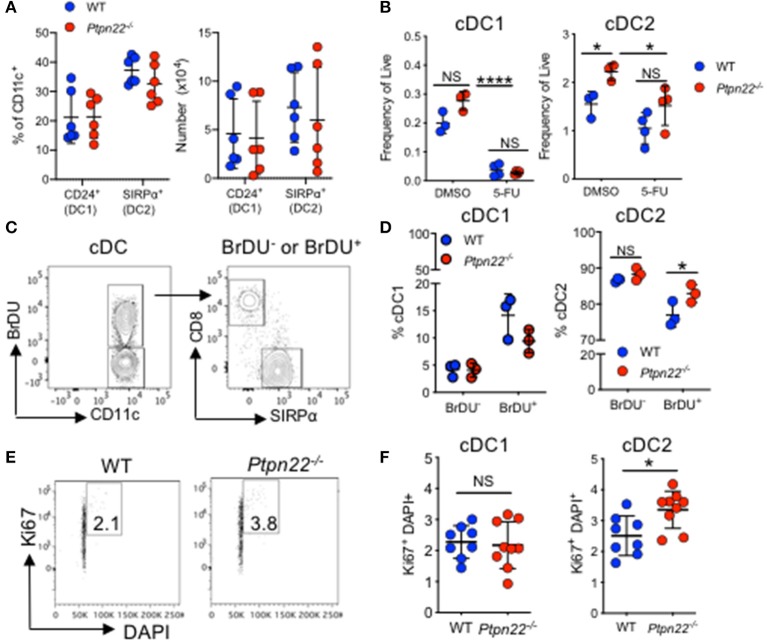
PTPN22 regulates DC2 proliferation. **(A)** Bone marrow from wild type (WT) or *Ptpn22*^−/−^ mice cultured in the presence of Flt3L for 8 days (Flt3L-BMDC). At day 8 the proportion and number of CD24^+^ cDC1 and SIRPα^+^ cDC2 were determined by flow cytometry. *N* = 6 mice per genotype from 6 independent experiments. **(B)** The frequency of live splenic cDC1 and cDC2 from WT and *Ptpn22*^−/−^ measured 3 days after i.v immunization with 5-flurouracil or DMSO control. *N* = 3–4 mice per group. **(C,D)** The percentage of splenic cDC1 and cDC2 within BrDU^−^ and BrDU^+^ populations within BrDU treated WT and *Ptpn22*^−/−^ mice. **(C)** Representative flow plots of analysis, quantified in **(D)**. *N* = 3 mice per genotype. **(E,F)** Ki67 and DAPI expression within splenic cDC1 and cDC2 subsets from WT and *Ptpn22*^−/−^ spleens. **(E)** Representative flow plot analysis and quantified in **(F)**. *N* = 8 mice per genotype. **(A,B,D,F)** Each point represents an individual mouse; bars represent mean and standard deviation. NS = not significant, **(A–F)** ^*^p < 0.05, determined by unpaired *T*-test. NS = not significant, ^*^p < 0.05.

Homeostasis of cDC in SLOs is controlled by multiple factors including differentiation of bone marrow precursors in response to Flt3L, duration of cDC survival, proliferation of incoming precursor cDCs, and the turnover of a small subset of cDCs within SLOs (1). We therefore aimed to examine how PTPN22 might regulate cDC2 development. Firstly, we observed no PTPN22 dependent difference in bone marrow or splenic common DC precursors (CDP) or preDC cells, indicating that PTPN22 operated post pre-cDC development (Supplementary Figures 3A–C). Secondly, as cDC1 and cDC2 are dependent on Flt3L for their differentiation we tested if PTPN22 controls Flt3L dependent cDC2. We observed similar Flt3R expression on *ex vivo* WT and *Ptpn22*^−/−^ cDC1 and cDC2 (Supplementary Figures 3D–E), as well as similar concentrations of serum Flt3L *in vivo* (Supplementary Figure 3F). To compare Flt3L dependent cDC2 development, we cultured WT and *Ptpn22*^−/−^ bone marrow *in vitro* with Flt3L. However, no significant changes in cDC2 development were observed (Figure 3A). We then assessed if PTPN22 altered cDC2 survival by comparing the expression of survival genes *Bcl2, Bim*, and *Trim2* in FACS sorted cDC2. Once again we observed no differences between WT and *Ptpn22*^−/−^ cDC2 (Supplementary Figure 3G). Furthermore, no differences were observed between splenic WT and *Ptpn22*^−/−^ cDC2 acquiring an apoptotic phenotype (Annexin V^+^) as a consequence of a 24-h culture *in vitro* (Supplementary Figure 3H). Based on these data, we reasoned that differences in cell survival were unlikely to be a major mechanism mediating cDC2 expansion in *Ptpn22*^−/−^ mice.

### PTPN22 Negatively Regulates cDC2 Proliferation

Having excluded a role for PTPN22 in regulating DC precursor development, Flt3L dependent differentiation or cDC2 survival, we next addressed if PTPN22 might control cDC2 proliferation. To test this hypothesis, we examined the *in vivo* effects of 5-flurouracil (5-FU), a pyrimidine analog that inhibits cell proliferation (46). Previous reports have demonstrated that 3 days post-5-FU administration DC populations are significantly reduced within the spleen, indicative of rapid turnover of DCs *in vivo* (47). The difference in cDC2 expansion between WT and *Ptpn22*^−/−^ mice was abrogated 3 days after treatment with 5-FU, demonstrating that PTPN22 mediated expansion of cDC2 is indeed dependent on proliferation (Figure 3B). Consistent with our data using 5-FU, administration of thymidine analog BrDU, which incorporates into proliferating cells, demonstrated that the *Ptpn22*^−/−^ cDC2 population was significantly expanded within the BrDU^+^ (proliferating), but not the BrDU^−^ (non-proliferating) population (Figures 3C,D). Furthermore, Ki67 and DAPI staining confirmed enhanced proportions of cDC2 undergoing cell cycling (Figures 3E,F and Supplementary Figure 3I). Likewise, cell cycle analysis of competitive bone marrow chimeras 3 weeks post transfer also demonstrated a significant increase in the proportion of cycling *Ptpn22*^−/−^ cDC2 when compared to WT controls (Supplementary Figure 3J). Taken together, these data support the notion that PTPN22 controls cDC2 homeostasis by restricting cDC2 proliferation.

### *Ptpn22*^619W^ Confers Expansion of ESAM^+^CD4^+^ cDC2s

**Figure 4:**
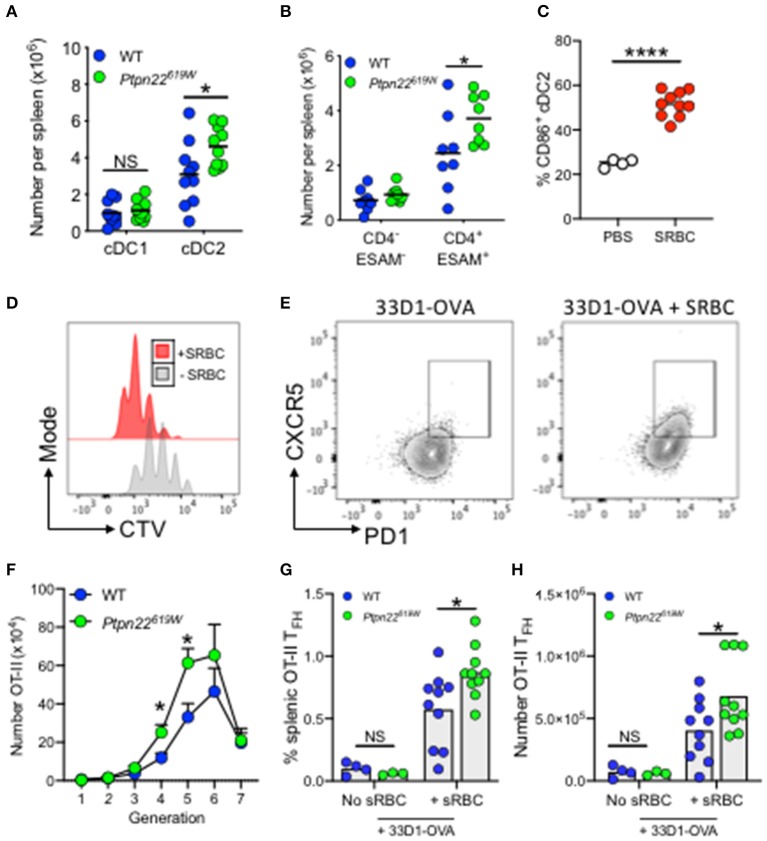
*Ptpn22*^619*W*^ conferred cDC2 expansion enhances T cell proliferation and T_FH_. **(A,B)** Spleens of 2–4 months age matched WT and *Ptpn22*^619*W*^ mice were evaluated for cDC subsets by flow cytometry **(A)** number of cDC1 and cDC2 per spleen **(B)** number of ESAM^−^ vs. ESAM^+^ cDC2 per spleen. **(A,B)** *N* = 10 mice per genotype. **(C)** Mice were immunized i.v. with PBS or SRBC and after 4 h splenic cDC2 were assessed for cell surface CD86 expression by flow cytometry. *N* = 4 or 10 mice/group. **(D–H)** CD45.1^+^CD4^+^ OT-II T cells were transferred i.v into recipient mice. The following day mice received i.v 33D1-ovalbumin in the presence or absence of sheep RBC (SRBC). After 3 days CD45.1^+^ CD4^+^ Vα2Vβ5^+^ T cells were evaluated for CTV dilution and CXCR5^+^PD-1^+^ T follicular helper cell (T_FH_) by flow cytometry. Representative plots of CTV dilution **(D)** and T_FH_ induction **(E)** in the presence or absence of SRBC. **(F–H)** OT-II proliferation and T_FH_ induction within WT and *Ptpn22*^619*W*^ recipient mice determined by flow cytometry. **(F)** The number of proliferating CD45.1^+^ CD4^+^ Vα2Vβ5^+^ OT-II T cells. **(G)** The frequency of CD45.1^+^ Vα2Vβ5^+^CD4^+^PD-1^+^CXCR5^+^ T_FH_ per spleen. **(H)** The number of CD45.1^+^ Vα2Vβ5^+^CD4^+^PD-1^+^CXCR5^+^ T_FH_ per spleen. **(F–H)** *N* = 10 mice/genotype. Each point represents an individual mouse, bars represent mean; error bars represent s.e.m. **(A–H)** ^*^p < 0.05, ^****^p < 0.0001 determined by unpaired *T*-test.

The human *PTPN22*^*R*620*W*^ polymorphism is one of the highest-ranking genetic risk factors for the development of multiple autoimmune diseases outside MHC loci (38, 48). Given that PTPN22 regulates the expansion of cDC2s, we set out to examine whether the autoimmune associated variant was capable of mediating similar effects. To address this, we enumerated splenic cDC subsets in mice expressing the R620W ortholog, *Ptpn22*^619*W*^. In comparison to *Ptpn22*^619*R*^, mice carrying *Ptpn22*^619*W*^ also displayed expansion of splenic cDC2s, which, like *Ptpn22*^−/−^ mice, occurred specifically within the ESAM^+^CD4^+^ DC2 subset (Figures 4A,B). Furthermore, the magnitude of cDC2 expansion was similar to *Ptpn22*^−/−^ mice when compared to WT (1.5-fold vs. 2-fold respectively). This demonstrated that the autoimmune associated PTPN22 variant is also capable of regulating cDC2 homeostasis, operating as a loss-of-function mutant in this context.

### *Ptpn22*^619W^ Enhances T Cell Proliferation and Generation of T_FH_

*PTPN22*^*R*620*W*^ is a risk allele associated with multiple autoantibody associated autoimmune diseases. Splenic cDC2 are essential initiators of T_FH_ differentiation, leading to GC formation, and high-affinity antibody production (18, 49). Interestingly, with age *Ptpn22*^−/−^ and *Ptpn22*^619*W*^ mice develop spontaneous GC, and enhanced serum IgG levels (33, 43, 50). Accordingly, we addressed whether expansion of splenic cDC2 in *Ptpn22*^619*W*^ mice was sufficient to alter cDC2 dependent T cell activation and T_FH_ induction *in vivo*. To evaluate cDC2 dependent antigen specific responses *in vivo* we administered 33D1-OVA conjugates to selectively target the cDC2 subset (Supplementary Figure 4) (8). WT and *Ptpn22*^619*W*^ mice received CD45.1 OT-II CD4^+^ T cells and were immunized with 33D1-OVA in the presence of SRBCs to promote cDC2 activation (Figure 4C), and potentiate OT-II proliferation and T_FH_ responses (Figures 4D,E) (18, 49). When compared to WT recipients, *Ptpn22*^619*W*^ cDC2 expansion was sufficient to enhance OT-II proliferation *in vivo* (Figure 4F). Furthermore, we observed that the proportion and number of splenic OT-II T_FH_ that develop within *Ptpn22*^619*W*^ recipients was significantly enhanced compared to WT following 33D1-OVA/SRBC immunization (Figures 4G,H). Our previous investigations demonstrate that Ptpn22 is dispensable for antigen uptake and presentation (51). Furthermore, the *in vitro* data presented here indicate that differences in OT-II proliferation are due to altered cDC2 homeostasis (specifically the number of cDC2 cells), rather than a cell intrinsic difference in Ptpn22 variant cDC2. When total, unmanipulated splenic cDC are FACS isolated (preserving the cDC2 expansion observed *in vivo*) and co-cultured with OT-II in the presence of 33D1-OVA, *Ptpn22*^−/−^ are able to potentiate OT-II T cell proliferation (Supplementary Figure 4B). Conversely, when cDC2 are FACS isolated (normalizing cDC2 numbers) and cultured in the same manner, WT and *Ptpn22*^−/−^ cDC2 are capable of inducing OT-II proliferation to the same extent. Furthermore, no difference in OT-II proliferation is observed when co-cultured with FACS isolated cDC1 in the presence of DEC205-OVA (Supplementary Figure 4D). Together, these data reveal that *Ptpn22*^619*W*^ mediated cDC2 expansion is sufficient to deregulate T cell activation in response to non-self-antigens, and is likely to contribute to the promotion of T_FH_ responses *in vivo* alongside previously reported T cell and B cell intrinsic effects (33, 50).

## Discussion

Here, we provide evidence that the autoimmune disease-associated phosphatase PTPN22 is a regulator of cDC2 homeostasis. Loss-of-function mutants of PTPN22 result in cDC2 expansion through mechanisms that are DC intrinsic, enhancing cDC2 proliferation. Thus, PTPN22 appears to be a selective regulator of cDC2 homeostasis. Furthermore, cDC2 expansion conferred by the autoimmune associated *Ptpn22*^619*W*^ variant resulted in aberrant cDC2 dependent T_FH_ induction *in vivo*. Our data therefore uncover a novel mechanism by which T_FH_ expansion, first reported in *Ptpn22* deficient mice, may be underpinned by a specific cDC2 phenotype.

The precise mechanisms by which Ptpn22 mediates selective expansion of cDC2 remains to be determined. However, we now know that *Ptpn22* deficiency mediates the expansion of ESAM^+^CD4^+^ cDC2 (Figure 1E), a cDC2 subset that is known to be dependent on LTβR for their proliferation (15). Furthermore, splenic *Ptpn22*^−/−^ cDC2 were more proliferative *ex vivo* (Figures 3B–F). Consistent with our data, *Lt*β*r*^−/−^ splenic cDC2s are less proliferative, resulting in decreased cDC2 numbers (31). Indeed, our preliminary experiments indicate that Ptpn22 may control LTβR mediated cDC2 proliferation. LTβR agonist treatment of *Ptpn22*^−/−^ Flt3L-BMDC increased cDC2 numbers compared to WT (Supplementary Figures 5A,B), whilst no difference in cell surface LTβR expression was observed on *ex vivo* or *in vitro* generated Flt3L BMDC (Supplementary Figures 5C–F). In addition, phenotypes described in *Relb*^−/−^ mice, further support the hypothesis that PTPN22 may regulate LTβR dependent cDC2 proliferation. LTβR activates canonical pathway and non-canonical NFκB signaling, resulting in RelB translocation (52). Within *Relb*^−/−^ mice, there is a severe reduction in splenic and LN resident (but not migratory) cDC2, from as early as 3 weeks of age, whereas cDC1 numbers are unaffected (53), and Flt3L dependent DC development is unaffected by *Relb*^−/−^. In addition, despite lamina propria DC being dependent on Notch2 and LTβR signaling, *Relb*^−/−^ does not affect CD11b^+^ DC subsets at this location (15, 53). In keeping with these reports, we observed no difference in lamina propria CD11b^+^ DC subsets between WT and *Ptpn22*^−/−^ mice (Supplementary Figure 6). Therefore, in both *Relb*^−/−^ and *Ptpn22*^−/−^ mice the same cDC2 populations are disrupted, being decreased in *Relb*^−/−^ and increased in *Ptpn22*^−/−^, with both occurring within a 3-weeks time frame. In contrast, due to differences in the specific cDC2 phenotypes reported, our data do not support a role for PTPN22 in regulating IRF4 (54), KLF4 (10), or NOTCH2 (12, 15) dependent cDC2 development. As such, our data are consistent with a model whereby PTPN22 may function to negatively regulate LTβR signaling, limiting RelB translocation to control cDC2 homeostasis.

One question that our data raise, is why and how Ptpn22 selectively regulates cDC2 homeostasis? One explanation might be the differential expression of Ptpn22 in DC subsets, which is substantially higher in cDC2 than cDC1 or pDC (ImmGen); implying that the effects of Ptpn22 deficiency are likely to be greatest in cells with the highest expression. An alternate explanation is that Ptpn22 regulates signaling pathways specifically required for cDC2 but not cDC1 development. In line with this, our data demonstrate no defect in pathways required by both cDC1 and cDC2, since both precursor cDC development (Supplementary Figures 3A–C) and Flt3L responsiveness (Supplementary Figure 3A) remain intact in *Ptpn22*^−/−^ mice. Conversely, proliferation was enhanced specifically in Ptpn22^−/-^ cDC2 population (Figure 3), indicating that Ptpn22 may operate to restrain cDC2 proliferation in response to signals required for cDC2 turnover. One factor known to mediate the proliferation of a small subset of cDC2 is LTβR, and Ptpn22 may be involved in regulating this proliferative signal *in vivo*. Alternatively, Ptpn22 may regulate the signaling of an as yet to be identified pathway that is also required for cDC2 proliferation. Further detailed investigation is required to uncover the mechanistic basis for the pathway(s) regulated by Ptpn22 in this context.

*PTPN22*^*R*620*W*^ is one of the strongest autoimmune disease associated genetic risk factors. We demonstrate that cDC2 homeostasis is disrupted in mice that express the ortholog of the human autoimmune associated variant (Figures 4A,B). To our knowledge, this is the first description of cDC homeostasis being regulated by an autoimmune associated genetic risk allele. Our data therefore provide a link between genetic and environmental risk and the breakdown of immune tolerance that leads to autoimmunity. Changes to cDC homeostasis have been described within autoimmune diseases for which *PTPN22*^*R*620*W*^ is a risk factor. In type 1 diabetes the effector phase of murine type I diabetes is characterized by cDC2 expansion (55). Furthermore, in humans with rheumatoid arthritis (RA), cDC2 are decreased within the blood, but expanded within the lymph nodes (56), and the RA synovium is enriched with RelB^+^ DCs (57). Together, suggesting an association between the enhanced presence of cDC2s within SLOs and tissue and the risk of developing autoimmune disease. Therefore, a failure to maintain cDC homeostasis, as conferred by the PTPN22 risk allele, may be a factor altering the downstream immune responses that ultimately lead to autoimmunity.

Although we accept that the difference between WT and *Ptpn22* variant cDC2 populations may appear modest, over the lifetime of a human, these changes could have significant functional impact over the decades that precede autoimmune disease onset. In the context of non-self cDC2-targeted antigen, *Ptpn22*^619*W*^ dependent expansion of cDC2 was sufficient to enhance T cell proliferation and T_FH_ expansion following GC promoting SRBC stimulation (Figures 4F,G). Activation of ESAM^+^ cDC2 is one of the earliest events in splenic GC formation leading to high-affinity antibody production (18, 49). With aging, PTPN22 mutant mice develop many hallmarks of autoimmunity including increased effector T cells, activated B cells and higher immunoglobulin and autoantibody titres (33, 43). Furthermore, humans carrying the PTPN22^620W^ variant have an increased risk of developing autoimmune diseases that are almost exclusively associated with autoantibody production (38, 48). PTPN22 has been reported to regulate T_FH_ (50) cells, GC formation, and antibody production (33) in part via T cell and B cell intrinsic effects. Although our data do not address self-reactivity, they do indicate that changes conferred by *Ptpn22*^*R*619*W*^ altering cDC2 homeostasis, could, alongside T and B intrinsic effects, also contribute to perturb the GC reaction over time. Age is an important risk factor for autoimmune disease onset due to declining immune competence and impaired immune tolerance check points (58). In keeping with this concept, cDC2 expansion was potentiated in aged *Ptpn22*^−/−^ mice (Supplementary Figure 1E) and as such changes to the balance of activating: inhibitory cells *in vivo* could alter cDC2 dependent responses that trigger the breaking of immune tolerance. Future work will be required to confirm the link between early expansion of cDC2 and autoimmunity.

In summary, our findings uncover PTPN22 as a selective regulatory checkpoint required to maintain cDC2 homeostasis, and suggest that early perturbation of DC homeostasis may be a trigger for the onset of autoimmunity.

## Data Availability Statement

All datasets generated for this study are included in the article/Supplementary Material.

## Ethics Statement

The animal study was reviewed and approved by Animal Welfare and Ethical Review Body (AWERB) King's College London.

## Author Contributions

HP designed research, performed experiments, analyzed data, and wrote the manuscript. FC, CC, AM, and GC performed experiments and analyzed data. JB analyzed data and contributed to writing the manuscript. XD, DR, and RZ developed and contributed vital mouse models. DD developed and provided vital 33D1 and DEC-205-OVA reagents. PG and AC conceived and funded the project, contributed to data analysis, and wrote the manuscript. All authors reviewed the manuscript.

### Conflict of Interest

The authors declare that the research was conducted in the absence of any commercial or financial relationships that could be construed as a potential conflict of interest.
