# Supplementary material for: Phosphatase PTPN22 Regulates Dendritic Cell Homeostasis and cDC2 Dependent T Cell Responses
Source: Front Immunol. 2020 Mar 4;11:376. doi: 10.3389/fimmu.2020.00376 (PMC7065600; doi:10.3389/fimmu.2020.00376)
Supplement: Supplementary file 1 [file Presentation_1.pdf]

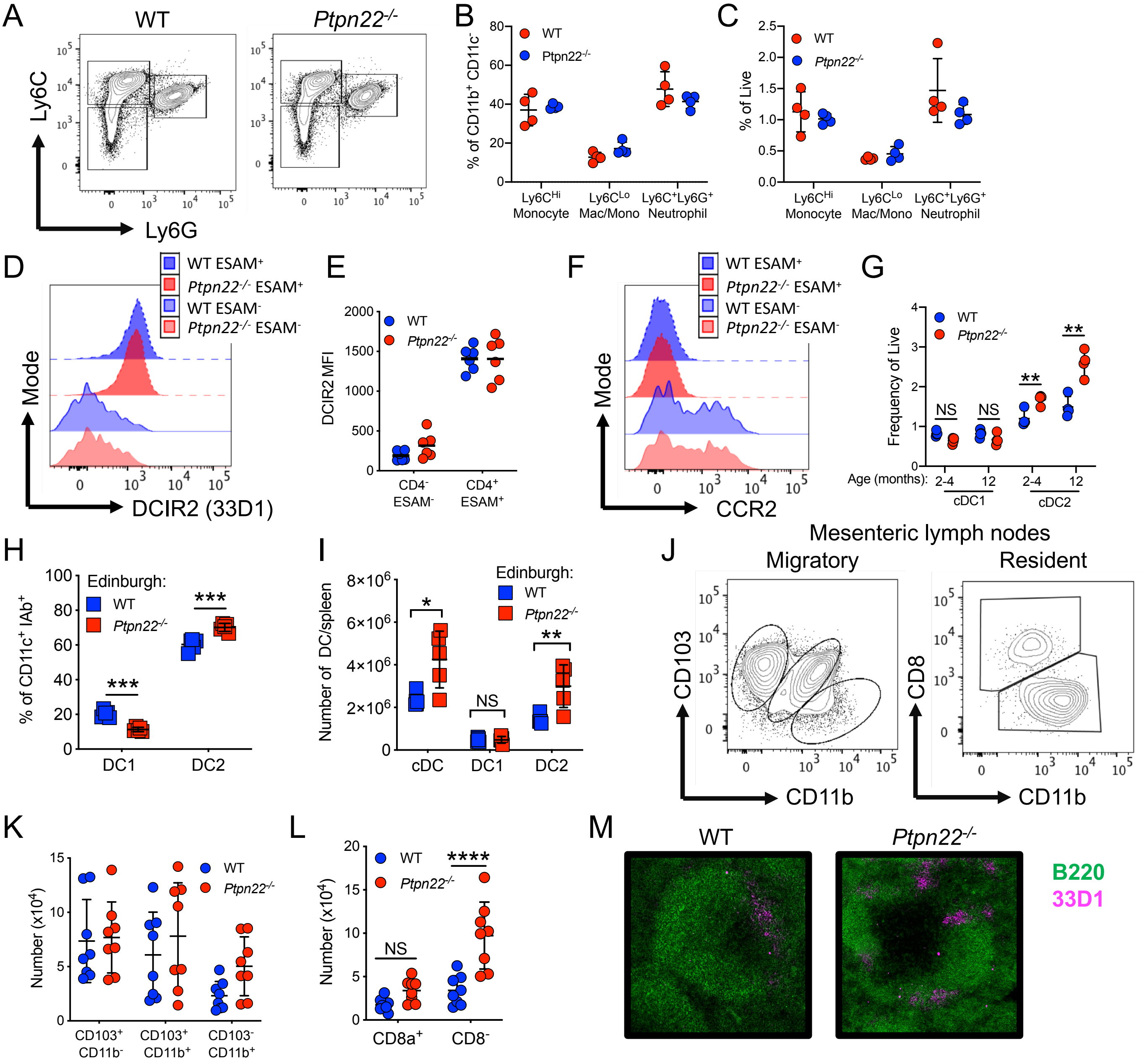

Supplementary Figure 1. PTPN22 regulation of cDC2 homeostasis. (A-C) WT and *Ptpn22*$^{-/-}$ spleens were assessed for CD11b^{+}CD11c^{-} monocyte (Ly6C^{+}Ly6G^{-}), macrophage (Ly6C^{lo}Ly6G^{-}) and neutrophil (Ly6C^{+}Ly6G^{+})subsets by flow cytometry. Representative gating strategy **(A)** and the frequency of CD11b^{+}CD11c^{-} **(B)** and live cells per cell type per spleen **(C)** was determined; N=4 mice/genotype. **(D,E)** Expression of DCIR2 (33D1) on CD4$^{+/-}$ESAM$^{+/-}$ DC subsets. **(D)** Representative flow cytometry staining **(E)** Median Fluorescent intensity N=6 mice/genotype from 2 independent experiments. **(F)** Representative flow cytometry staining of CCR2 expression on CD4$^{+/-}$ESAM$^{+/-}$ DC subsets. N=3 mice/genotype. **(G)** Spleens of 2-4 month and >1 year old WT and *Ptpn22*$^{-/-}$ were assessed by flow cytometry for the frequency of cDC1 and cDC2 ; N=4 mice/genotype. **(H-I)** WT and *Ptpn22*$^{-/-}$ spleens from three 2 month and two 6 month old mice bred and maintained in an independent animal facility (Edinburgh University), were assessed for **(H)** proportion of cDC1 and cDC2 **(I)** number of cDC1 and cDC2; N=5 mice/genotype. **(J-L)** Mesenteric lymph node migratory and resident cDC subsets within 2-4 month WT and *Ptpn22*$^{-/-}$ mice. Gated on: singlet, live, lin^{-} CD11c^{+} MHCcII I-Ab^{High} (migratory) or CD11c^{+} MHCcII I-Ab^{Int} (resident) cDC. **(J)** Representative flow cytometry plots of migratory and resident cDC subsets **(K)** number within migratory cDC subset **(L)** number within resident cDC subsets; 8 mice/genotype from 2 independent experiments. **(M)** WT and *Ptpn22*$^{-/-}$ spleens (2 months) were sectioned and stained for 33D1 and B220 and imaged by confocal microscopy. Representative images of 3 mice per group. In graphs each point represents an individual mouse; bars represent mean, NS = not significant, *p<0.05, **p<0.01, ***p<0.001, ****p<0.0001 determined by unpaired T-test.

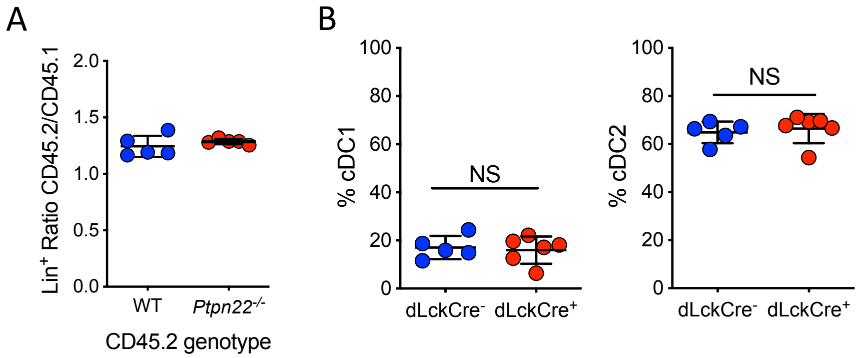

Supplementary Figure 2. cDC2 homeostasis is normal in T-cell restricted *Ptpn22*$^{-/-}$. (A) Lethally irradiated CD45.1/2 recipient mice received a 1:1 ratio of WT CD45.1 : WT or *Ptpn22*$^{-/-}$ CD45.2 bone marrow (i.v). After 8 weeks spleens of recipient mice were evaluated for cDC subsets and the ratio of CD45.1:CD45.2 within Lineage^{+} (CD3, CD19, B220, NK1.1, Ly6C/G) determined by flow cytometry. The ratio of CD45.1:CD45.2 within Lineage^{+} gate calculated relative to the input ratio**.** N=5 mice/genotype, one experiment of two. **(B)** Spleens of dLckCre^{-} and dLckCre^{+} (*Ptpn22*$^{-/-}$*)* mice were assessed cDC1 and cDC2 subsets by flow cytometry. N=5-6 mice/genotype. Each point represents an individual mouse; bars represent mean, NS = not significant, *p<0.05 determined by unpaired T-test.

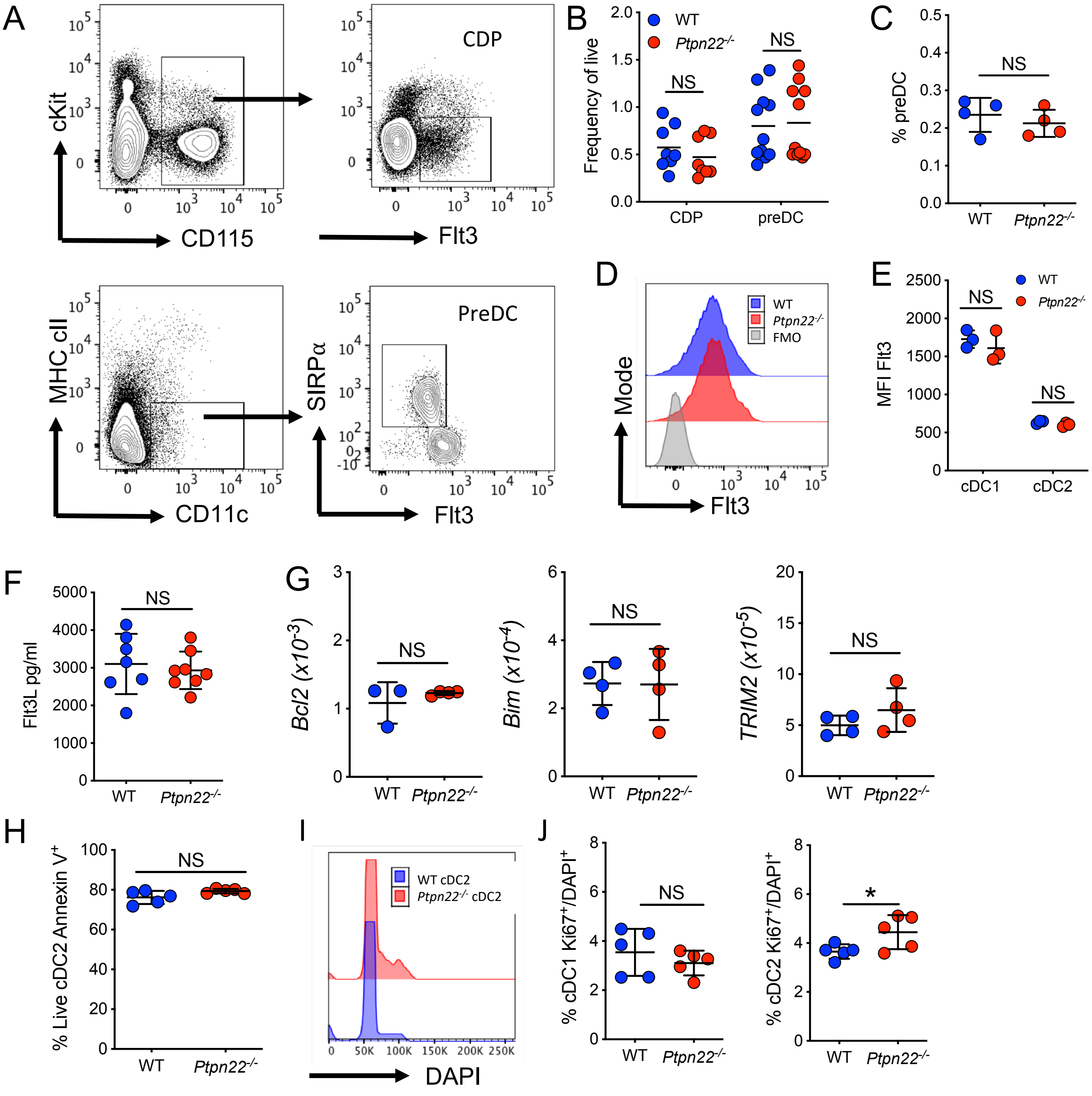

Supplementary Figure 3. PTPN22 regulates DC2 homeostasis post preDC development. (A-C) The presence of common DC precursors (CDP - $CD115^{+}cKit^{-}Flt3^{+}$) and preDC ($CD11c^{+}$, MHCcII $I\text{-}A^{b-}$, $SIRP\alpha^{-}$, $Flt3^{+}$) were determined within bone marrow and spleens from wild type (WT) and *Ptpn22*$^{-/-}$ mice by flow cytometry gating on: live, singlet, lin^{-} ($CD3^{-}CD19^{-}Ly6G/C^{-}Ter119^{-}NK1.1^{-}B220^{-}$). **(A)** example flow cytometry gating of CDP (top) preDC (bottom) **(B)** Percentage of bone marrow CDP and preDC; 8-11 mice/genotype **(C)** Percentage of splenic preDC; 4 mice/genotype. **(D-E)** Cell surface Flt3R expression on splenic cDC1 and cDC2 subsets; N=3 mice per group. Representative flow cytometry plots displayed in **(D)** and median fluorescent intensity quantified in **(E). (F)** WT and *Ptpn22*$^{-/-}$ mouse serum was analyzed for Fl3L by ELISA; N=7-8 mice/ genotype. **(G)** *Bcl2*, *Bim*, and *Trim2* mRNA expression in FACS sorted cDC2s from wild type (WT) and *Ptpn22*$^{-/-}$ mice, normalized to expression of *Gapdh*. **(H)** Proportion of live cDC2 Annexin V^{+} apoptotic cells from digested spleens after 24 hours incubation at 37°C; N=5 mice/genotype. **(I)** Flow cytometric histogram of DAPI expression within splenic cDC1 and cDC2 subsets from WT and *Ptpn22*$^{-/-}$ spleens. **(J)** Spleens from WT and *Ptpn22*$^{-/-}$ competitive bone marrow chimeras were assessed 3 weeks post bone marrow transfer for $Ki67^{+}DAPI^{+}$ cDC1 and cDC2 subsets within CD45.2 transferred cell by flow cytometry; N=5 mice/genotype. **(A-J)** Each point represents an individual mouse; bars represent means. NS = not significant, $^{*}p<0.05$ determined by unpaired T-test.

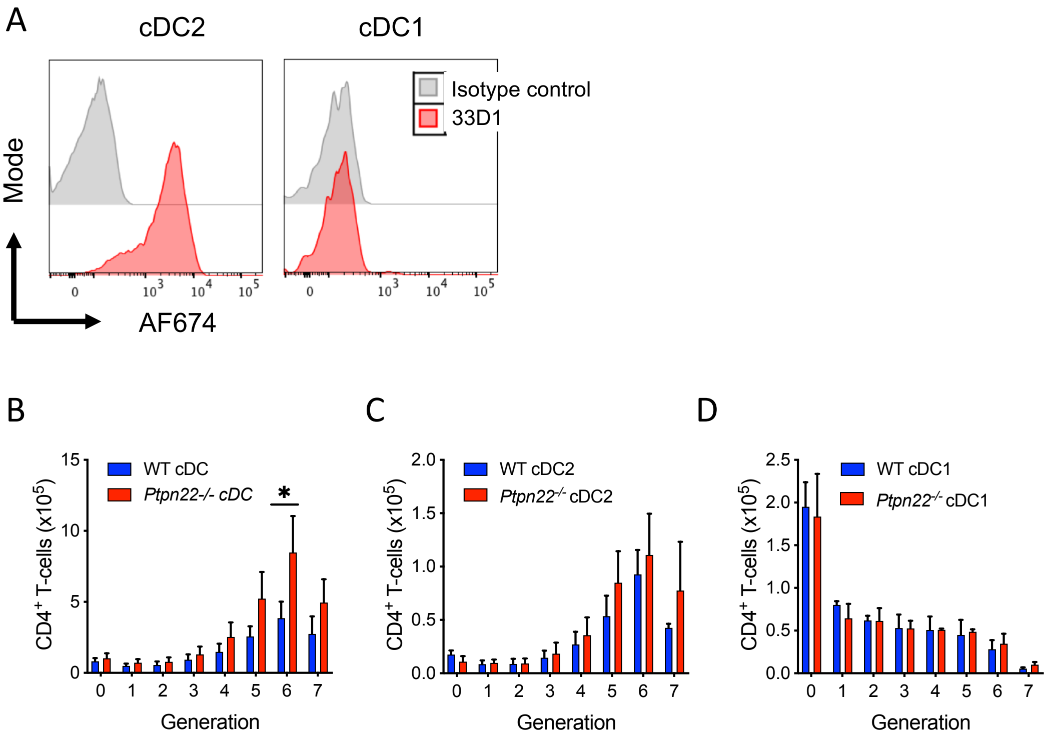

Supplementary Figure 4. Enhanced cDC2 number rather than altered function promote CD4 T cell proliferation. (A) Expression of 33D-1 expression on splenic cDC2 and cDC1 2 hours after immunization with 33D1-AF647 or isotype control-AF647 determined by flow cytometry. **(B-D)** Total cDC **(B)** or cDC2 **(C)** or cDC1 **(D)** were FACS isolated from spleens of 2-4 month WT and Ptpn22-/- mice and co-cultured with cell trace violet (CTV) labelled OT-II $CD4^+$ T cells in the presence of 33D1-ovalbumin **(B,C)** or anti-DEC205-ovalbumin **(D)** for 6 days. The number of OT-II $CD4^+$ T cell per generation was determined within each CTV division by flow cytometry. Data are of 4 independent experiments.

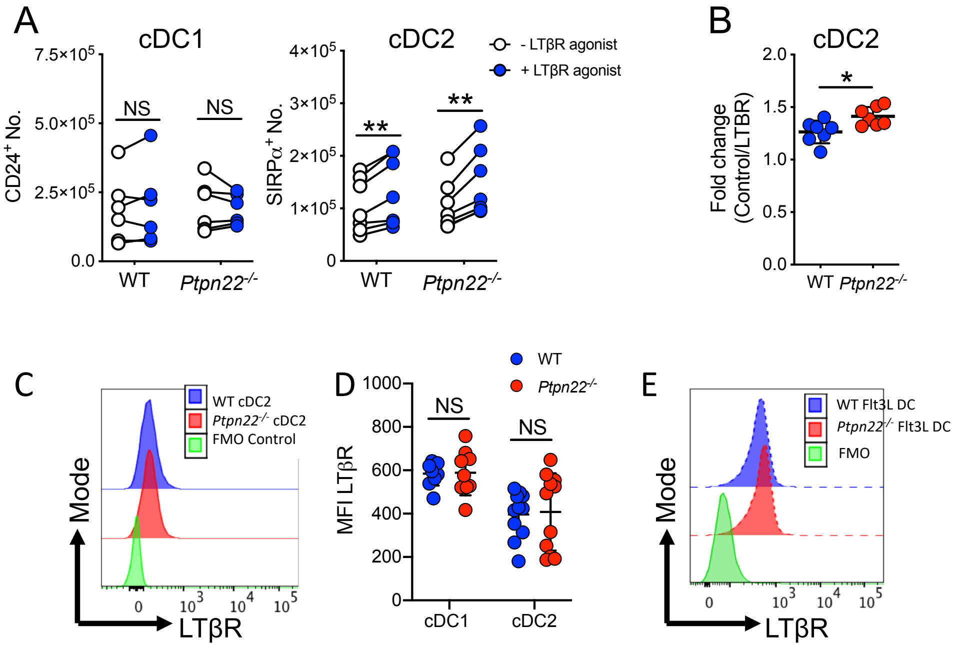

Supplementary Figure 5. Enhanced LTBR mediated cDC2 expansion by *Ptpn22*$^{-/-}$ Flt3L BMDC (A) Number of cDC1 and cDC2 generated following 48h LTβR agonist stimulation of day 8 WT and *Ptpn22*$^{-/-}$ Flt3L-BMDC. N= 6-8 independent experiments; each point is derived from the mean of triplicate technical repeats for an individual mouse. **(B)** Fold change of LTβR agonist response relative to Flt3L alone **(**white circles/blue circles from **(A - cDC2)**. Each point represents an individual mouse; bars represent mean and standard deviation. Paired T-test. NS = not significant, *p<0.05, **p<0.01. **(C, D)** Cell surface LTβR expression on splenic cDC1 and cDC2 subsets (**C**) representative flow cytometry plot and median fluorescent intensity quantified in **(D).** N=10-11 mice/genotype. **(E)** Cell surface LTβR expression on Day 8 Flt3L-BMDC. Each point represents an individual mouse; bars represent means. NS = not significant, *p<0.05 determined by unpaired T-test.

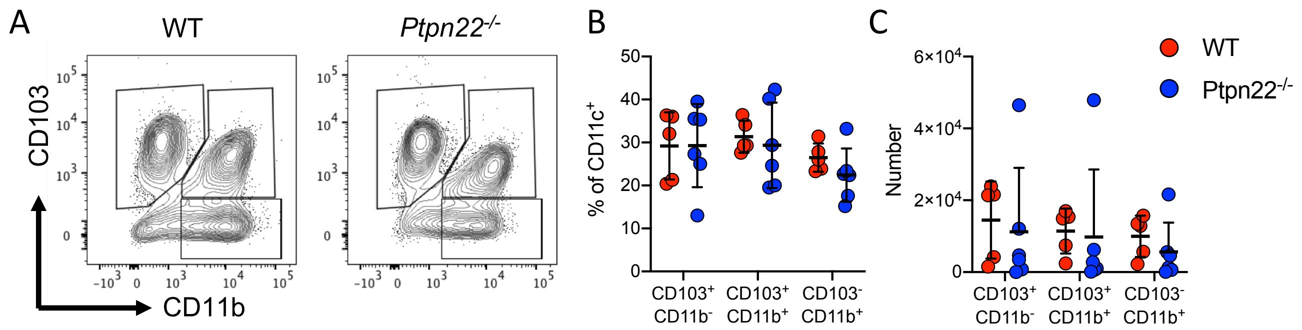

Supplementary Figure 6. Lamina propria DC subsets are similar between WT and *Ptpn22*$^{-/-}$ mice number. Lamina propria of 2 month old WT and Ptpn22-/- mice with peyers patches removed were digested and subsequently assessed for DC subsets by flow cytometry. **(A)** Representative staining of lamina propria CD11c^{+} CD103 vs CD11b subsets. The % (B) and number **(C)** of CD103^{+}CD11b^{-}, CD103^{+}CD11b^{+} CD103^{-} CD11b^{+} within the CD11c gate. Data are of 5 mice per genotype performed over two independent experiments.
